# Maximal Strength, Sprint, and Jump Performance in High-Level Female Football Players Are Maintained With a Customized Training Program During the COVID-19 Lockdown

**DOI:** 10.3389/fphys.2021.623885

**Published:** 2021-02-26

**Authors:** Sigurd Pedersen, Dag Johansen, Andrea Casolo, Morten B. Randers, Edvard H. Sagelv, Boye Welde, Andreas Kjæreng Winther, Svein Arne Pettersen

**Affiliations:** ^1^School of Sport Sciences, Faculty of Health Sciences, UiT The Arctic University of Norway, Tromsø, Norway; ^2^Department of Computer Science, Faculty of Natural Sciences and Technology, UiT The Arctic University of Norway, Tromsø, Norway; ^3^Department of Bioengineering, Imperial College London, London, United Kingdom; ^4^Department of Biomedical Sciences, University of Padova, Padua, Italy; ^5^Department of Sport Sciences and Clinical Biomechanics, Faculty of Health Sciences, University of Southern Denmark, Odense, Denmark

**Keywords:** resistance training, sprint, soccer, counter movement jump, squat, COVID-19

## Abstract

**Introduction:**

The COVID-19 outbreak with partial lockdown has inevitably led to an alteration in training routines for football players worldwide. Thus, coaches had to face with the novel challenge of minimizing the potential decline in fitness during this period of training disruption.

**Methods:**

In this observational pre- to posttest study involving Norwegian female football players (18.8 ± 1.9 years, height 1.68 ± 0.4 m, mass 61.3 ± 3.7 kg), we investigated the effects of a prescribed home-based and group-based intervention, implemented during the COVID-19 lockdown, on maximal muscular force production and high velocity variables. Specifically, maximal partial squat strength one repetition maximum (1RM), counter movement jump (CMJ) and 15 m sprint time were assessed 1 week prior to the lockdown and 12 weeks after the onset of lockdown. We also collected training content and volume from the prescribed training program and self-reported perceived training quality and motivation toward training.

**Results:**

We observed no change in 1RM [pretest: 104 ± 12 kg, posttest: 101 ± 11 kg (*P* = 0.28)], CMJ height [pretest: 28.1 ± 2.3 cm, posttest: 26.8 ± 1.9 (*P* = 0.09)], and 15 m sprint time [pretest: 2.60 ± 0.08 s, posttest: 2.61 ± 0.07 s (*P* = 0.52)].

**Conclusion:**

Our findings suggest that a prescribed home-based and group-based intervention with increased training time devoted to strength, jump, and sprint ability, and regulated to obtain a sufficient infection control level is feasible and effective to preserve strength, jumping, and sprinting abilities of high-level female football players during a ∼ 3-month period of a pandemic-induced lockdown.

## Introduction

In 2019 and 2020, the COVID-19 outbreak resulted in country lockdowns where both the general population and athletes were exposed to unexpected behavioral restrictions (e.g., social distancing, closure and/or limitation of non-essential activities such as gyms and training grounds, and ultimately, self-isolation). Strict quarantine rules were introduced following national/international travel, after direct exposure to the virus or if showing COVID-19 symptoms. Consequently, athletes were enforced to cancel and/or postpone their competitions and to abruptly adjust their training routines (Sarto et al., 2020). In Norway, these regulations were imposed for all sports and were introduced during football teams’ pre-season preparations. This led to a rapid shift in training plans and training practice with for example, some teams prescribing home-based training for their players (Sarto et al., 2020).

Football fitness includes both aerobic and anaerobic-capacity, and explosive muscle actions (Bangsbo et al., 2006). For example, dynamic muscle strength such as partial squat one repetition maximum (1RM) is suggested to reflect functional strength of football players (Wisløff et al., 1998), which is associated with muscular power (Stølen et al., 2005) and the ability to perform football specific actions (Wing et al., 2020). Mimicking the movement patterns in football during training was challenged during the lockdown period, which may have led to declines in football-specific physical fitness (Mohr et al., 2020).

One of the main intentions of the pre-season period in football is to optimize physical performance including jumping and sprinting ability, and maximal strength (Rønnestad et al., 2011). However, how these abilities are affected when the pre-season is unexpectedly interrupted, is mostly unknown. Several researchers have suggested negative effects of self-isolation following the COVID-19 lockdown (Mohr et al., 2020; Sarto et al., 2020), suggesting that it may result in lower training volume and quality, and in turn, decreased physical fitness (Girardi et al., 2020; Sarto et al., 2020). Indeed, a number of studies have reported reduced physical activity (Xiang et al., 2020; Zheng et al., 2020) and training hours during the COVID-19 lockdown (Mon-López et al., 2020; Zinner et al., 2020), and there are already findings of decreased cycling performance in cyclists (Muriel et al., 2020) and reduced hamstring strength in football players (Moreno-Pérez et al., 2020). However, some have presented the potential of maintaining physical fitness in multidisciplinary sports such as football, by performing circuit-based training (Latella and Haff, 2020). This was recently shown in a male football team, where jump height was preserved following 15 weeks of isolated training (Cohen et al., 2020).

Longer periods without strength training (12 weeks) may lead to reduced strength of 7–12% in strength trained individuals (Mujika and Padilla, 2000). Importantly, small quantities of training can attenuate the strength loss following complete training cessation in high levels athletes (García-Pallarés et al., 2010). Our planned data collection involving female football players was abruptly interrupted by the pandemic. Thus, we had the opportunity to investigate whether a change in prescribed training designed to limit COVID-19 infection during lockdown (home-based, group based, and without normal football play), could preserve 1RM partial squat strength, counter movement jump (CMJ) and 15 m sprint time. To the authors’ knowledge, the effect of COVID-19 related training adjustments on strength and strength derivatives is only available in male football players (Cohen et al., 2020; Moreno-Pérez et al., 2020). Thus, the aim of our study was to assess the effects of a prescribed unsupervised 12-week home- and group-based training program without gym facilities on 1RM partial squat strength, CMJ and 15 m sprint time in female high-level football players during a period without full contact football training.

## Materials and Methods

### Design, Subjects, Procedure, and Questionnaires

**FIGURE 1:**
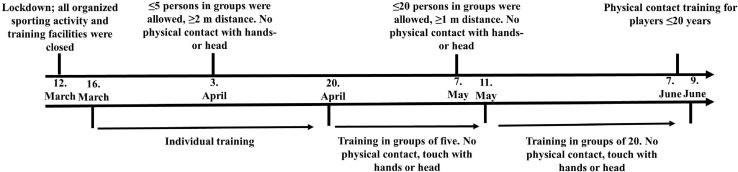
Timeline of COVID-19 regulations affecting the team in the current study. The chart displays both the governmental and football associations’ regulations (upper side of the line) and the teams’ prescribed training to these regulations (lower side of the line). Pretest was carried out prior to the first date in the scheme, while posttest was performed immediately after the last date.

Our study is a longitudinal 12-week observational study with a pretest posttest design. Two female football teams playing at level three in Norway were originally invited to another study, whose main aim was to investigate the association between high-force/power tests and physical performance derived from tracking data during football match play. Since the COVID-19 lockdown was imposed by national authorities 1 week following pretest start, and 1 day prior to pretest the second team (*n* = 13) (Figure 1), the team that completed the lab tests (*n* = 13) involved in that study were invited to participate in a new data collection (posttest) 12 weeks after the first visit to the laboratory. Four players had left Tromsø for personal reasons during the lockdown period, thus nine players attended the follow-up measurements and were eligible for this study (Table 1). The lockdown was introduced 4 weeks prior to the planned start of the competitive season for the team. Data for eight players is reported for the force data acquisition in the CMJ test and eight players for 1RM, due to incomplete force data recording in the CMJ for one player at pretest and a hand injury at posttest for another player. Eight players are included in the dexa-scan data. All the other variables are reported for pre- and posttest for all nine players. The training plan information was retrieved from the team’s coaches (Table 2).

**TABLE 1:** Baseline participant characteristics(mean ± SD).

Age (year)	18.8 ± 1.9
Body mass (kg)	61.3 ± 3.7
Height (m)	1.68 ± 0.4
Lean mass (kg)	44.5 ± 2.0
Bone mass (kg)	2.66 ± 2.0
Body fat (%)	24.88 ± 0.04
Fat mass (kg)	14.9 ± 3.5
Leg lean mass (kg)	15.4 ± 8.6

*Body composition is measured by a dual-energy X-ray absorptiometry machine (dexa-scan) (Lunar Prodigy; GE Medical Systems, Buckinghamshire, United Kingdom).*

**TABLE 2:** Changes in prescribed training from pre- to posttest, before and during lockdown.

	**During normal pre-season training before lockdown**	**During lockdown**	**%-change**
Total training (min)	29177	23347	−20
Football training (min)	20755	95106	−54
Strength training (min)	2827	4039	43
Speed and jump training (min)	613	2619	333
Endurance training, running (min)	00	4539	NA
Individual training (sessions)	0.30.5	2.02.0	567
Group/team training (sessions)	3.60.7	1.81.7	−50

*Weekly training prescribed by the coaches to their players during the spring 2020. The data is presented as average minutes ± SD.*

According to the Helsinki declaration, all participants were informed of the potential benefits, risks, and procedures of the study, both orally and in writing, before signing an informed consent. The original study and the data storage protocol was approved by the Norwegian Center for Research Data (NSD) (approval reference number: 989024), and approved our changed aim and a new data collection period in June (correction reference number: 768380), without any further ethical approval per institutional and national guidelines for research on sport and exercise science.

At pretest, the players answered one custom-made question about their experience with strength training in the squat exercise with answering option 1, No experience; 2, Some experience (<1 year); and 3, A lot of experience (>1 year). Two months after posttest in June 2020, the players received an individual custom-made questionnaire about their pre-season training habits before and during the lockdown. This consisted of six questions where the first reflected their adherence to the training prescribed by the coaches during the lockdown, in a bipolar five-unit Likert-scale. The second and third question were about their perceived quality of training and motivation to training, respectively, answered on a bipolar three-unit Likert scale. These three questions were first addressed for the pre-season period prior to the lockdown, and subsequently for the period after the lockdown. The questionnaire was designed by two researchers (SP and SAP), and later discussed by additional two researchers (BW and TH) where the final version was developed with consensus from all four researchers.

Prior to pretest, all the participants underwent 3–4 supervised familiarization sessions to the partial squat exercise over 2 weeks, in order to perform the movement safely and technically sound (Ploutz-Snyder and Giamis, 2001). All tests and familiarization sessions started with the same standardized warm-up routine consisting of 5 min of self-selected low intensity cycling on an ergometer bike (Pro/Trainer, Wattbike Ltd., Nottingham, United Kingdom) and followed by 5 min low intensity running of self-selected speed in the gym. The participants were recommended to not perform strenuous physical exercise 24 h prior to testing. Pretests of physical fitness (e.g., 1RM, CMJ, and 15 m sprint) were conducted in the laboratory between 17:00 and 19:00 on two separate occasions, separated by 3 days. During the first day, the sprint tests were carried out first followed by the CMJ test; thereafter half of the players underwent the dexa-scan. On the second day, the players underwent a 1RM test, followed by a dexa-scan for the second half of the players.

### Strength

The participants underwent a 1RM test in the partial back squat exercise for the assessment of maximal dynamic strength. An Olympic barbell (T-100G; Eleiko, Halmstad, Sweden) was used for both familiarization and the main experimental testing. During the 1RM trials, the participants were instructed to lift the bar from the rack, step one step back, go slow into the descending phase of the movement, followed by a maximal intended velocity during the concentric phase. The participants initiated the concentric phase as response to an orally “go” by the researcher measuring the 90° knee angle at the knee joint with a goniometer (Pedersen et al., 2019). The same researcher measured knee angle for all the participants during both pre- and posttest. The participants warmed up with 10 repetitions by lifting the bar (20 kg), followed by 10 repetitions of the participants’ perceived ∼50% 1RM. The starting 1RM attempt consisted of one repetition at a high load, which the participants knew they could manage. Each following attempt was completed with 5–10 kg additional load until failure. All players had a minimum of 3 min rest between each lift. The highest load lifted by the participants was defined as their 1RM.

### Sprint

Prior to the sprint test, in addition to the general standardized warm up, the participants performed three 15 m strides at their subjective effort of 85–90% of maximal acceleration speed. Thereafter, the participants performed three, 15 m sprints with 3 min rest between each attempt. The sprint times were measured by single-beam electronic photocells (ATU-X, IC control AB, Stockholm, Sweden) mounted to the floor and walls. The starting photocell was placed 20 cm above the ground, while the 5, 10, and 15 m photocells were placed 100 cm above the ground. Self-initiated, the players started the sprint in a static position placing their front foot 30 cm behind the starting line. The fastest 5, 10, and 15 m split times were included in the analyses.

### Counter Movement Jump

Standing on the force platform (Hur-Labs, ALU4, Finland), the participants were instructed to perform a CMJ with the aim to jump as high as possible, with the hands fixed on the hips during the entire movement. Force data were recorded with bespoke software (Force platform software suite, HURlabs oy, Kokkola, Finland). The software calculates jump height as the center of mass displacement calculated from the force developed and measured body mass. Each player performed two trials with a minimum 3 min rest between the two trials. The highest jump was taken for analysis.

### Statistical Analyses

The Shapiro–Wilk test and visual inspection of Q–Q plots were used to assess normality distribution of data. For 1RM, CMJ, and 15 m sprint times we used Student paired sample *t*-tests to determine the change from pre- to posttest. For training and questionnaire data, non-parametric tests were used for analyses as these variables were considered non-normally distributed. For the questionnaire data (e.g., training adherence, quality, and motivation), we used a non-parametric signed rank tests to assess the direction of the data from pre-post lockdown (Roberson et al., 1995). All data are presented as mean ± standard deviation (SD), or 95% confidence intervals (CI). Alpha level was set to 0.05. The Statistical Package for the Social Sciences (SPSS, Version 26, IBM, Armonk, NY, United States) was used for the statistical analyses.

## Results

Six of the players reported to have some experience with strength training using the squat exercise, while the three remaining players reported to have a lot of experience.

**FIGURE 2:**
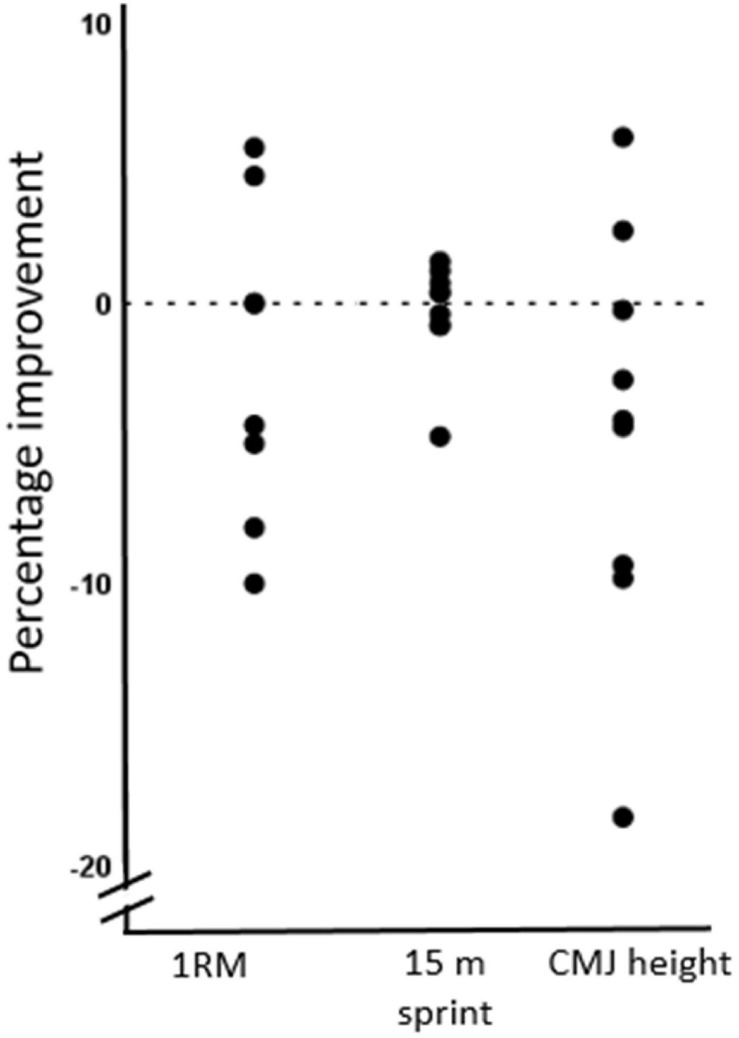
Percentage changes for each individual participant from pre- to posttest for absolute one repetition maximum in the partial squat exercise (1RM) (kg), 15 m sprint time (s) and counter movement jump (CMJ) height (cm).

The pre- and posttest results are presented in Table 3, with the individual percentage changes illustrated in Figure 2. No significant changes were observed for absolute- and relative 1RM partial squat strength (*P* > 0.05), CMJ jump height (*P* > 0.05), force production variables in the CMJ test (all *P* > 0.05), or in 15 m sprint times (*P* > 0.05, in all cases) from pre- to posttest.

**TABLE 3:** Changes in selected parameters from pre- to posttest.

	**Pre (mean ± SD)**	**Post (mean ± SD)**	**Change (mean and 95% CI)**	***P*-value**
**Partial squat 1RM**				
1RM (kg)	10412	10111	3(3;8)	0.28
1RM (kg⋅m_b_^–1^)	1.690.24	1.650.23	0.03(−0.05;0.12)	0.39
**15 m sprint test**				
5 m (s)	1.030.04	1.040.05	0.01(−0.05;0.03)	0.52
10 m (s)	1.860.05	1.880.05	0.01(−0.04;0.01)	0.28
15 m (s)	2.600.08	2.610.07	0.01(−0.04;0.03)	0.68
**CMJ**				
Jump height (cm)	28.12.3	26.81.9	1.4(−0.26;2.98)	0.09
Takeoff velocity (m s^–1^)	2.340.10	2.290.08	0.05(−0.03;0.13)	0.17
Maximum force (N)	127470	126573	9(−58;77)	0.75
Maximum power (W)	2665167	2605109	60(−96;216)	0.40
Flight time (ms)	49026	48518	5.6(−15;26)	0.57
Average power (W)	62889	59176	36(−8;81)	0.10
Average force (N)	76337	74737	16(−8;40)	0.15

*CMJ, counter movement jump; 1RM, one repetition maximum. *P*, the *P*-value from the paired sample *t*-test from pre- to posttest.*

**FIGURE 3:**
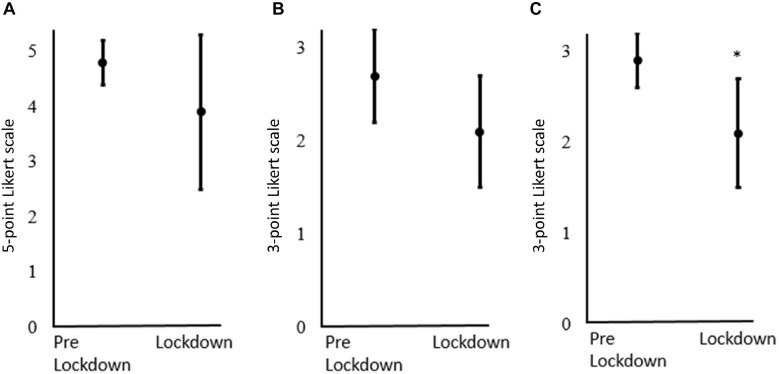
**(A)** Perceived adherence to proportion of the prescribed training plan [*y*-axis, 1. to a little degree (0–20%), 2. to some degree (21–40%), 3. approximately half (41–60%), 4. to a large degree (61–80%), 5. almost all (81–100%)]. **(B)** Perceived degree of motivation for training (*y*-axis, 1. highly motivated, 2. average motivated, 3. low motivated). **(C)** Perceived level of quality of the training conducted (*y*-axis, 1. high quality, 2. average quality, 3. low quality). The figure displays the mean ± SD. ^∗^Indicates a significant difference (*P* < 0.05) from pre lockdown to lockdown, derived from the Signed rank test.

Change in perceived adherence to the prescribed training and perceived level of motivation toward training were both non-significant (*P* > 0.05), while perceived quality of training was significantly reduced from pre- to posttest (*P* < 0.05) (Figure 3).

## Discussion

Our study describes the effects on partial squat 1RM, CMJ, and sprint performance in female football players during a period of a global pandemic, which resulted in comprehensive adjustments of players’ training routines. Our main findings were that maximal strength, sprint time and jump performance did not decrease during lockdown.

Researchers have speculated that COVID-19 induced restrictions will lead to training cessation and consequently physiological detraining during (Girardi et al., 2020), where football players first trained in solitude, followed by a second stage in which training was performed in small groups with contact restrictions (Mohr et al., 2020). The team in our study had a total weekly training volume of ∼ 5 h prior and ∼ 4 h during the lockdown, which was a decreased total training time (∼1 h). This is less than the ∼ 3.5 h reduced training time recently reported for female football players in the three highest leagues in Spain during the lockdown (Mon-López et al., 2020). The participants in our study tended to perform more strength training during the lockdown, potentially compensating for the loss of sports specific training during this period. This increase in strength training time did not increase strength, jump, or sprint performance, but rather maintained it. Importantly, our results reflect the prescribed training from the coaches, and not the reported training performed by the players, as in the study by Mon-López et al. (2020). However, their initial training volume was probably much higher than in the current study, as their self-reported weekly training pre-lockdown was 12 h. The training volume in our study is much lower than for elite players. Self-reported training data from the first and second placing team in the highest division in Norway last season, showed that the players trained 9.2 ± 2.5 weekly hours during the preseason (unpublished results).

We did not expect that reduced football training *per se* would lead to a marked strength loss, as football training do not seem to influence maximal strength (Rønnestad et al., 2008). We rather expected that no access to the team’s- and commercial gyms might have a negative influence on maximal strength during the COVID-19 period. Recently, one study found that a group of semi-professional male football players reduced hamstring muscle strength following 25 days of home confinement due to the COVID-19 lockdown (Moreno-Pérez et al., 2020). However, these findings are not directly comparable as the muscles, exercise modality and muscular contraction type (Nordic hamstring, an eccentric contraction exercise) were different for the strength test in the study by Moreno-Pérez et al. (2020) than in our study. Eccentric strength is more susceptible to decline compared to other neuromuscular factors (Mujika and Padilla, 2001). This may explain the discrepancy between our study and the study by Moreno-Pérez et al. (2020), as the partial squats in our study emphasize concentric strength.

Highly strength trained athletes are more susceptible to decrements in strength following training restriction compared to less trained (Rønnestad et al., 2011). The prescribed training by the team in our study prior to the lockdown consisted of little time devoted to strength training (Table 2). Hence, pretest 1RM was likely not influenced by systematic maximal strength training in the players in our study. For example, our study’s players’ 1RM was similar to the baseline 1RM partial squat strength in another study of female football players, which increased their 1RM squat strength by 30% following 5 weeks of strength training (Pedersen et al., 2019). However, although the players in our study reported to have some experience with the squat exercise, it may be that their exercise modalities were ineffective or non-specific, thus their maximal partial squat strength was relatively low compared to the levels expected following systematic strength training (Pedersen et al., 2019).

The sprint time for 15 m accompanied by split times at 5 and 10 m did not change during the lockdown. A study by Sporis et al. (2011) showed that starters in official matches improved sprint performance more than non-starters over a season. Specific small-sided games are typically used as training drills during normal football training. Small-sided games are shown to both provoke high maximal acceleration distance during play (Ade et al., 2014) and improve sprint performance (Hammami et al., 2018). However, sprinting is an uncomplicated movement pattern to carry out outside the football field where especially short sprints can be trained almost anywhere without the use of equipment, and consequently lead to improvements in acceleration (Spinks et al., 2007), or sprint performance maintenance, as found in our study, being in line with earlier findings (Mujika et al., 2009). The sprint training effect is usually not superior when a supervising coach is included compared with individual unsupervised sprint training (Haugen et al., 2015). Thus, sprint-training effect solely relies on the players themselves, where the players in our study performed sufficient amounts to preserve sprint performance. The prescribed volume of sprint and jump training time showed a tendency to increase during the lockdown (Table 2), and may have compensated for the reduced specific football training.

The CMJ height was preserved during the lockdown. This is in line with recent findings in male football players (Cohen et al., 2020). Jump performances as during heading situations are observed in large sided football games (Owen et al., 2014). In order to follow the regulations by the authorities, both large sided and small-sided games were not performed by the team in the current study during the lockdown. With total training cessation, jump height decrements are reported following 4 weeks in adult males (Izquierdo et al., 2007), but not following 3 weeks in adolescent males (Gavanda et al., 2020). Maximal power is likely better preserved than maximal force (Bosquet et al., 2013). Importantly, the reduction is shown to be more profound as the training cessation duration increases, where maximal force declines are reported following 3 weeks of training cessation (Bosquet et al., 2013).

Others have found reduced jump height after an off-season training program (Koundourakis et al., 2014). The same is shown for sprint performance (Clemente et al., 2021). However, if the players are given an exercise plan during the off-season with gym sessions implemented, sprint and jump maintenance is possible (Requena et al., 2017). In our study, the players had not access to gyms, and were thus no able to implement proper maximal strength training in line with previous recommendations (Rønnestad et al., 2008; Helgerud et al., 2011). However, they still managed to implement a feasible training regimen sufficient to preserve strength levels. In total, the preserved strength, jump, and sprint performance during the COVID-19 lockdown in the team in our study is likely an effect of adhering to a well-designed physical training program, which according to our questionnaire was well implemented by the participants. For players of this level, strength training in the gym or full contact football trainings are not necessary to maintain maximal strength, running speed or jump ability.

### Limitations

The main limitation in the current study is the low sample size, which increases the possibility of a statistical type 2 error, and such a consequence cannot be conclusively ruled out. However, to our knowledge this is the first study to report pre- and posttest prior and following the COVID-19 lockdown in female football players. Due to the uncontrolled design of our study, it should be considered descriptive and thus report changes in acceleration and jump height over the COVID-19 training period. Unfortunately, this design does not allow for causality inference (i.e., why we observed no change in performance). This information is highly relevant for football coaches. Second, although the training was described by the coaches, we do not know whether the players adhered to the training regimen, which we measured in retrospect with a subjective rating on the Likert scale.

## Conclusion

Maximal partial squat strength, CMJ, and sprint performance were preserved in female football players during a 12-week period of absence from normal football training and gym facilities during the COVID-19-induced lockdown. Thus, all the variables reflecting maximal and rapid muscular force production remained unchanged, likely due to adherence to a well-designed home-based and group-based training program by the team’s coaches.

### Future Steps and Recommendations

Furthermore, as football is a complex sport in its requirements for different physical aspects, coaches should consider breaks like those that we have experienced due to COVID-19 as opportunities, and prioritize endurance, speed and power training in lieu of specific football training.

## Data Availability Statement

The raw data supporting the conclusions of this article will be made available by the authors, without undue reservation.

## Ethics Statement

Ethical review and approval was not required for the study on human participants in accordance with the local legislation and institutional requirements. The patients/participants provided their written informed consent to participate in this study.

## Author Contributions

SP: in charge of the writing process, conceptualization and design, and data collection. SAP and DJ: conceptualization and design, and critical manuscript revision. AW and ES: contribution to data collection, statistical analyses, and manuscript revision. BW: conceptualization and design, contribution to statistical analyses, and critical manuscript revision. MR and AC: contribution to critical manuscript revision. All authors contributed to final approval of the version to be published.

## Conflict of Interest

The authors declare that the research was conducted in the absence of any commercial or financial relationships that could be construed as a potential conflict of interest.
